# Aerogel-Like Material Based on PEGylated Hyperbranched Polymethylethoxysiloxane

**DOI:** 10.3390/polym15194012

**Published:** 2023-10-07

**Authors:** Kirill Borisov, Alexandra Kalinina, Aleksandra Bystrova, Aziz Muzafarov

**Affiliations:** 1 A.N. Nesmeyanov Institute of Organoelement Compounds, Russian Academy of Sciences, 119334 Moscow, Russia; borisov@ispm.ru (K.B.);; 2 Enikolopov Institute of Synthetic Polymeric Materials, Russian Academy of Sciences, 117393 Moscow, Russia

**Keywords:** polymethylsilsesquioxanes, hyperbranched polymers, nanogels, aerogels, microencapsulation, surfactant-free emulsions, hydrophilic–lipophilic balance

## Abstract

Aerogels are a class of materials that have gained increasing attention over the past several decades due to their exceptional physical and chemical properties. These materials are highly porous, with a low density and high surface area, allowing for applications such as insulation, catalysis, and energy storage. However, traditional aerogels, such as pure silica aerogels, suffer from brittleness and fragility, which limit their usefulness in many applications. Herein, we have addressed this problem by using organosilicon compounds, namely polymethylsilsesquioxane derivatives, for the synthesis of aerogel-like materials. Specifically, we have developed a novel approach involving surfactant-free synthesis of microcapsules from partially PEGylated hyperbranched polymethylethoxysiloxane. Due to the highly diphilic nature of these compounds, they readily concentrate at the oil/water interface in aqueous emulsions encapsulating oil droplets. During the subsequent condensation, the organosilicon precursor is consumed for hexane encapsulation (yielding hollow microcapsules) followed by the formation of a continuous condensed phase. Concurrently, methyl groups ensure the hydrophobicity of the resulting materials, which eliminates the need of using additional reagents for their hydrophobization.

## 1. Introduction

Aerogels hold immense potential for a wide range of applications across various domains. The vast potential for further development and refinement of aerogel-based materials makes them a subject of continued interest and exploration. They have found utility in areas such as sound insulation [1,2], thermal insulation [3,4], optical materials [5,6], as well as food industry [7] and numerous other fields. Among the diverse spectrum of materials capable of forming aerogel frameworks, such as carbon nanotubes [8,9,10], cellulose [11,12], polymer nanofibers [13], and natural substances [14,15,16,17], silica aerogels are among the most renowned. These materials were originally conceptualized by Kistler in 1931, and his groundbreaking work introduced the pioneering method of supercritical drying [18]. This method, which is now widely employed, facilitates the creation of aerogels with exceptionally low density and high porosity.

Silica and organosilicon aerogels have become a focal point of extensive research efforts, boasting a rich literature spanning multiple studies [19,20,21,22,23,24,25,26,27]. These studies explore a wide array of methodologies for their synthesis. Precursors employed in the fabrication of silicon–organic aerogels encompass a diverse range of substances. These include sodium silicate [19,20], methyltriethoxysilane [21], methyltrimethoxysilane [22,23,24], tetraethoxysilane [25], tetramethoxysilane [26], perfluoroalkylsilane [27], and many other silanes, often in combination with one another.

A crucial stage in the fabrication of aerogels is the drying process, as it plays a fundamental role in preserving the gel’s structure and, consequently, its physical and mechanical properties, as well as its porosity. There are three primary drying methods employed for their production.

The first of these methods is supercritical drying, which, while costly, proves highly effective. This technique is executed under elevated temperature and pressure conditions, inducing the transition of the liquid into a supercritical state. This transformation effectively negates the impact of capillary forces that might otherwise cause gel collapse [28]. Supercritical drying finds extensive use in the production of organosilicon aerogels [18,23,26].

The second method involves drying under ambient conditions [29,30,31]. This type of drying is notably straightforward to implement since it does not necessitate expensive or complex processes. However, it does impose limitations on the chemical composition of the gels. This is because, in cases where the gel’s pore surfaces are hydrophilic, the removal of water can lead to significant gel shrinkage, negatively impacting its properties. To mitigate this effect, hydrophobic silanes like trimethylchlorosilane, dimethyldichlorosilane, trimethylethoxysilane, methyltrimethoxysilane, and certain other silanes could be employed.

The third method is the freeze-drying of gels [20,32,33]. To execute this method, the liquid within the gel is frozen and then subjected to sublimation under vacuum conditions. This technique is more cost-effective compared to supercritical drying and can also prevent gel shrinkage. However, it does come with its limitations. The solvent’s crystallization during freezing can potentially compromise the integrity of the gel structure, leading to the formation of cracks.

An approach method for achieving a highly porous structure involves assembling specific micro-objects as building blocks, resulting in a hierarchically organized structure that combines high porosity with mechanical strength surpassing that of conventional aerogels. Güler et al. [34] have successfully applied this approach to create high-capacity adsorbents, and other researchers [35,36] have explored its potential in thermal insulation materials. Nevertheless, instances of this approach remain limited in the literature.

The basis of the current study is to further development of the concept of creating hollow particles using hyperbranched polyethoxysiloxane (PEOS) as a multifunctional inorganic matrix of dense globular shape [37,38,39]. Poly (ethylene glycol) derivatives of PEOS exhibit enhanced surface activity at the water–oil interface, enabling the production of nanoscale hollow silica particles [40,41]. Here, aerogel-like materials were prepared based on PEGylated hyperbranched polymethylethoxysiloxanes (PMEOS) in oil-in-water emulsions. The evolution of the PEGylated hyperbranched PMEOS in water involves polycyclization with the formation of a nanogel structure [42,43] and further assembly of the nanogel particles on the hexane–water phase boundary. During gelation, nanogel particles form crosslinked layers around hexane droplets, encapsulating them (forming hollow microcapsules) and a continuous condensed phase that binds the emerging microcapsules. The methyl groups confer hydrophobicity to the resulting materials, obviating the need for additional reagents and processes to make them hydrophobic.

## 2. Materials and Methods

### 2.1. Materials

Methyltriethoxysilane (99% Reachem, Moscow, Russia) was distilled under argon prior to use. Acetic acid (99% Component-Reaktiv, Moscow, Russia), and ethanol were dried by prolonged boiling, followed by distillation over P_2_O_5_ under argon. Sodium hydroxide (99% Component-Reaktiv, Moscow, Russia), aqueous solution of ammonia (25%, SIGMATEK, Khimki, Russia), poly(ethylene glycol) monomethyl ether (average molecular weight 350, ABCR, Karlsruhe, Germany), and hexane (99% Component-Reaktiv, Moscow, Russia) were used as received. Deionized water was used for all experiments.

Synthesis of hyperbranched polymethylethoxysiloxane (PMEOS). The synthesis was conducted using the methodology first described in [44]. A total of 267.5 g (1.5 moles) of methyltriethoxysilane (MTES) was dissolved in toluene to obtain 50% solution. The dissolution process was carried out at a temperature of T = 20 ± 5 °C for 5 min. An amount of 60 g (1.5 moles) of sodium hydroxide was added to the prepared solution. The mixture was vigorously stirred under an argon atmosphere until the sodium hydroxide particles disappeared. The resulting solution was diluted with toluene to a 25% concentration, and then 90 g (1.5 moles) of acetic acid were added dropwise. The mixture was stirred at a temperature of T = 20 ± 5 °C for 3 h. The prepared mixture was filtered using a Schott filter, and the solvent was removed using a rotary evaporator and a vacuum pump. The yield of the product was 136.1 g (87%).

PEGylation of hyperbranched PMEOS with a degree of ethoxy group substitution of 5, 10, 20 mol% (PMEOS-PEG-5, 10, 20) was conducted as follows: PMEOS was mixed with the calculated amount of PEG and stirred on an oil bath at a temperature of 135 °C for 6 h with the distillation of the generated ethanol. Subsequently, residual ethanol was removed by vacuum treatment (see Table 1 for loadings and yield).

PEGylation of hyperbranched PMEOS with a degree of ethoxy group substitution of 10 mol% (PMEOS-PEG-10) was conducted as follows: 40 g of PMEOS was mixed with 9 g of PEG and stirred on an oil bath at a temperature of 135 °C for 6 h with the distillation of the generated ethanol. Subsequently, residual ethanol was removed by vacuum treatment. The yield of the product was 47.5 g (99%).

PEGylation of hyperbranched PMEOS with a degree of ethoxy group substitution of 5 mol% (PMEOS-PEG-5) was conducted as follows: 40 g of PMEOS was mixed with 4.5 g of PEG and stirred on an oil bath at a temperature of 135 °C for 6 h with the distillation of the generated ethanol. Subsequently, residual ethanol was removed by vacuum treatment. The yield of the product was 43.7 g (99%).

Preparation of Aerogel. In 50 g of water, 1, 2, or 3 g of PMEOS-PEG was dispersed in 50 g of water. An amount of 1 g of hexane was added to this emulsion, and the mixture was stirred for 5 min at 700 rpm. Then, 2.5 g of ammonia was added, and stirring continued until gelation. The obtained gel was aged for a week and then subjected to freeze-drying for 48 h at a pressure of 0.08 Torr.

### 2.2. Methods

GPC Analysis. Gel-permeation chromatography (GPC) was performed on a chromatographic system consisting of a STAIER series II high-pressure pump (Aquilon, Nakhodka, Russia), a RIDK 102 refractometric detector (Czech Republic), and a JETSTREAM 2 PLUS column thermostat (KNAUER, Berlin, Germany). The temperature was controlled at 40 °C (±0.1 °C). Tetrahydrofuran was used as the eluent, the flow rate was 1.0 mL/min. A 300 × 7.8 mm column filled with Phenogel sorbent (Phenomenex, Torrance, CA, USA), particle size of 5 μm, and a pore size of 103 Å were used (passport separation range—up to 75,000 D). Recording and processing of data was carried out using UniChrom 4.7 software (Minsk, Belarus).

^1^H NMR Spectroscopy. ^1^H NMR spectra were acquired using a Bruker WP250 SY spectrometer with CDCl_3_ as the solvent.

IR spectroscopy. IR spectra were recorded on a Bruker Tensor 27 spectrometer in the ATR mode of 4 scans for each wave number in the range of 550–4000 cm^−1^.

Interfacial Tension Measurement. The interfacial tension (IFT) between the water and oil phases was determined using a Krüss spinning drop tensiometer at a temperature of 25 °C.

Contact Angle Measurement. Contact angle measurements were performed using the Krüss easy drop instrument.

Scanning Electron Microscopy. Scanning electron microscopy (SEM) was conducted using a JCM-6000 PLUS microscope equipped with an energy-dispersive spectrometer, operating at accelerating voltages ranging from 5 to 15 kV.

Transmission Electron Microscopy. Transmission electron microscopy (TEM) was performed using a JEM-2100F microscope.

Specific Surface Area Measurement. Nitrogen adsorption was measured using the dynamic adsorption–desorption method on a “Sorbi–MS” instrument (META, Russia) with helium as the carrier gas. The specific surface area of the materials was evaluated using the four-point BET method within a range of relative pressure (p/p_0_) of 0.06 to 0.2.

Mechanical Properties. Cylinder-shaped samples with dimensions of 35 mm × 24 mm (height × diameter) were loaded into a Mecmesin MultiTest 2.5-i instrument and subjected to uniaxial compression at 25 °C and a constant deformation rate of 1 mm/min.

Oil Absorption Capacity Measurement. An amount of 0.2 g of the sample was mixed with 10 g of sunflower oil, stirred for 30 min, and subsequently subjected to centrifugation at 3000 rpm for 15 min. After centrifugation, the oil was decanted, and the sediment was then weighed.

## 3. Results and Discussion

### Synthesis and Properties of PMEOS-PEG

**Figure 1:**
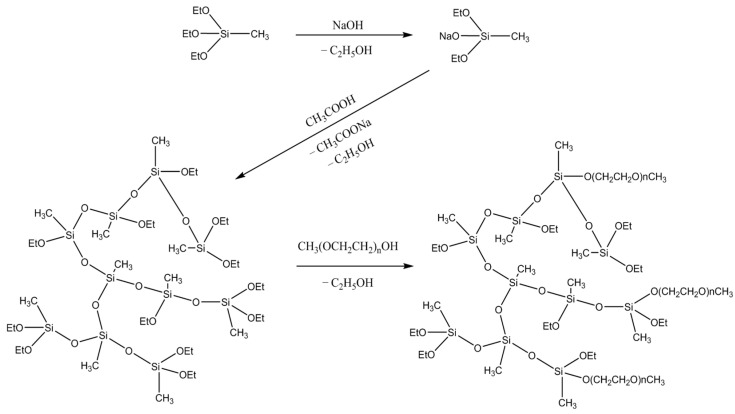
Synthesis of PMEOS-PEG.

**Figure 2:**
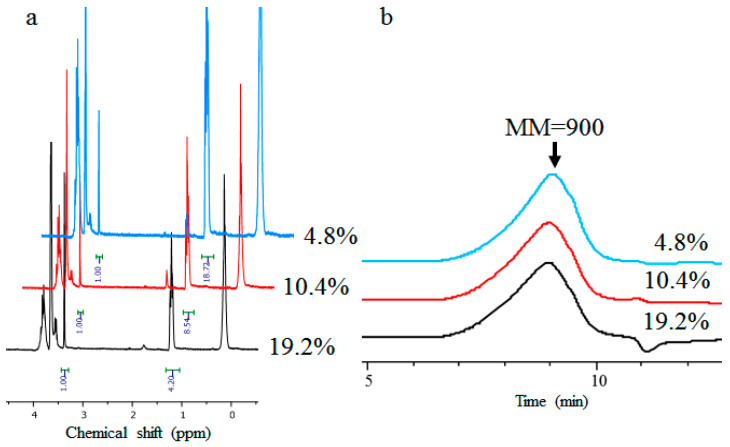
^1^H NMR spectra (**a**) and GPC curves (**b**) of PMEOS-PEG samples.

According to the scheme depicted in Figure 1, three samples of PMEOS-PEG were synthesized, containing 5, 10, and 20 mol% ethylene glycol substituents. ^1^H NMR data (Figure 2a) indicated that the quantity of substituted ethoxy groups is in good agreement with theoretical calculations, measuring 4.8, 10.4, and 19.2 mol%, correspondingly. Additionally, as demonstrated by GPC results (Figure 2b), all three PMEOS-PEG samples exhibit nearly identical molecular weight distributions.

To investigate the surface activity of the synthesized PMEOS-PEG, their solutions in toluene were mixed with water. The assessment of PMEOS-PEG surface activity at the water–toluene interface (Table 2) revealed that with increasing PMEOS-PEG concentration, the interfacial tension decreases. At the initial concentration of 10% of PMEOS-PEG-20 in toluene, the interfacial tension reaches a value of 5.2 mN/m, which is six times lower than the interfacial tension at the water-toluene interface.

**Figure 3:**
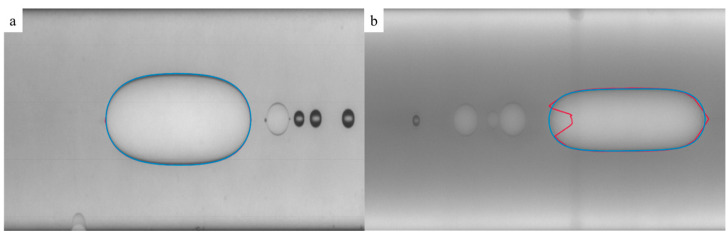
Hexane droplet in water in the presence of PMEOS-PEG-20 (PMEOS-PEG-20 content in water—0.1% (**a**) and 5% (**b**)).

Due to their highly diphilic nature, PMEOS-PEGs are soluble both in hydrophobic solvents and in water. To investigate their activity at the water–hexane interface, dispersions of PMEOS-PEG in water were mixed with hexane (Table 3, Figure 3). The most significant reduction in interfacial tension was logically observed for the dispersion of PMEOS-PEG-20 in water at the highest concentration (5%), where the interfacial tension value reached 1.9 mN/m (Figure 3). This provides the potential for the formation of hollow particles through PMEOS-PEG condensation on the surface of hexane droplets acting as templates. Hence, we hypothesized that these PMEOS-PEG condensation conditions would facilitate the production of hollow particles of minimal dimensions.

**Figure 4:**
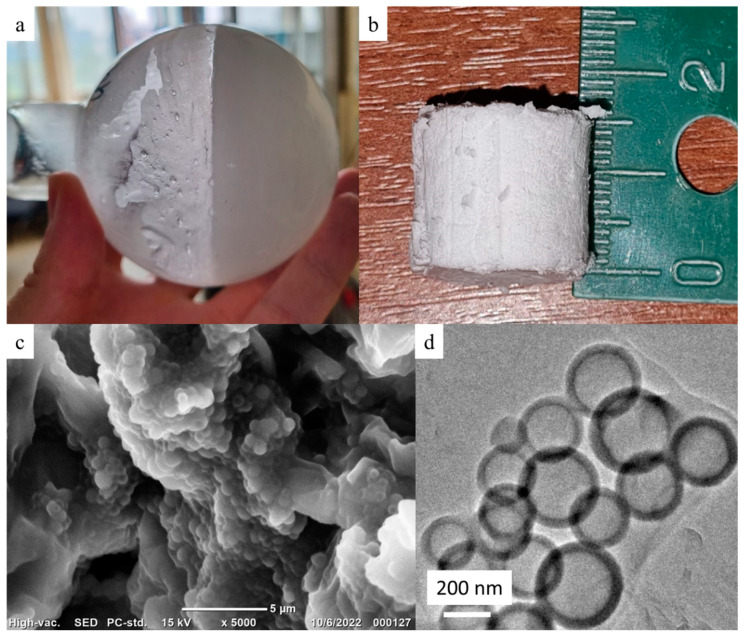
Gel in the flask (**a**), gel after freeze-drying (**b**), SEM image of Sample 2 after annealing (**c**), TEM image of Sample 2 after annealing (**d**).

**Figure 5:**
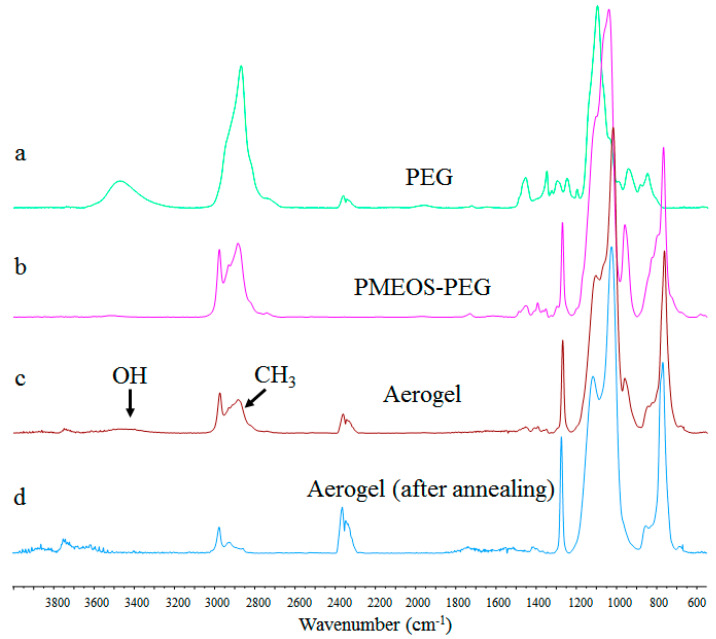
IR spectra of PEG (**a**), PMEOS-PEG-20 (**b**), aerogel sample after freeze-drying (**c**), aerogel sample after annealing at 200 °C for 2 h (**d**).

To produce aerogels, three PMEOS-PEG-20 to hexane mass ratios were employed: 1:1, 2:1, and 3:1 (designated as samples No. 1, 2, and 3, respectively), and the gelation of the prepared emulsions occurred after 11, 7, and 3 h, respectively. After aging for a week and freeze-drying for 48 h, a soft, elastic, yet crumbling material was obtained (Figure 4b). SEM and TEM images (Figure 4c,d) demonstrate that the hollow particles within the aerogel are polydisperse and range in size from 100 nm to 1.5 µm. The masses after freeze-drying were 0.79, 1.63, and 2.54 g, and the densities were 0.038, 0.04, and 0.059 g/cm^3^ for samples 1, 2, and 3, respectively. The contact angle values of water droplets on the surface of these materials were measured to be 120 ± 7°. These contact angle values are influenced by the presence of methyl groups within the gel composition. However, the presence of residual hydroxyl and ethoxy groups (Figure 5c) hinders the attainment of materials with greater hydrophobicity.

After annealing for 2 h at 200 °C, the aerogels lost a significant amount of their mass (Table 4), along with a decrease in their density. The reduction in these parameters indicates a substantial amount of unreacted ethoxy groups presented in the samples prior to annealing, which is confirmed by the decrease in the intensity of peaks around 2900 cm^−1^ corresponding to the vibrations of methyl components of the ethoxy groups (Figure 5c), and a small amount of hydroxyl groups (3350–3600 cm^−1^) (Figure 5d). It is important to emphasize here that such a significant loss of mass was accompanied with only 11.5% volumetric shrinkage (calculated from the dimensions of the samples), as the porous structure is established during the hexane encapsulation process. This fact is further confirmed by measurement of average specific surface area which increased after annealing by more than 100 times (see Table 4) from 1.25 m^2^/g for all samples before annealing.

**Figure 6:**
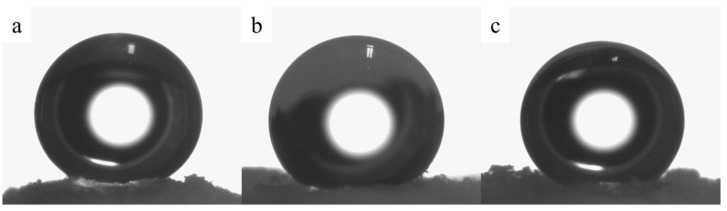
Water droplet on the surface of aerogel samples 1 (**a**), 2 (**b**), and 3 (**c**).

Upon the removal of residual ethoxy and hydroxyl groups, the contact angle reaches values of 140 ± 2° (Table 3, Figure 6) for annealed samples. These elevated contact angle values contribute to the aerogels’ excellent hydrophobicity, effectively averting the absorption of moisture from the surrounding environment and, consequently, preserving their physical and mechanical properties.

To determine the oil absorption capacity of the aerogel samples, we investigated their sorption properties with sunflower oil. The sorption results were as follows: 10.9 g of oil per 1 g of gel for sample 1, 13.1 g of oil per 1 g of gel for sample 2, and 11.4 g of oil per 1 g of gel for sample 3. These values align with the variations in specific surface area among the samples.

**Figure 7:**
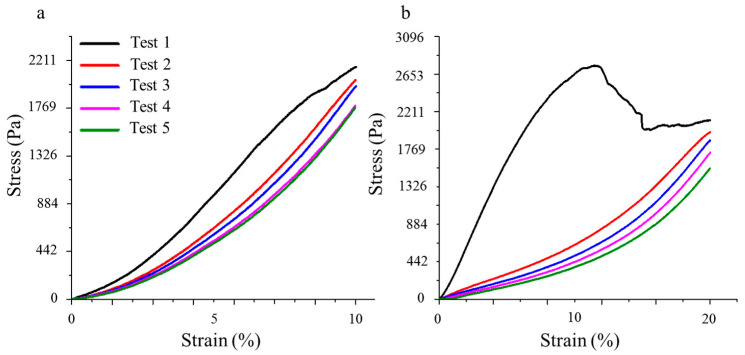
Stress–strain curves for sample compression by 10% (**a**) and 20% (**b**).

The mechanical properties of aerogel 2 were studied using compression strength measurement. During the experiments, the studied samples were gradually compressed at a rate of 1 mm/min, and each sample underwent five testing cycles. In the first experiment, a compression test was conducted on the sample at 10% of its height, repeated five times in a row (Figure 6a). The curves show that during the first test, the sample exhibits the best physical and mechanical properties. In the course of further testing, the load at which the sample deforms by 10% decreases from 2145 Pa to 1772 Pa by the fifth test. After each cycle, the aerogel relaxes to its initial dimensions. In the case of deformation tests at 20% (Figure 6b), partial destruction of the aerogel occurs in the very first experiment at a load of 2746 Pa. However, after removing the load, the material also returns to its initial dimensions (Figure 7). In subsequent tests, the aerogel demonstrates a significant decrease in physical and mechanical characteristics, as the stress required for 10% deformation decreases from 2653 Pa to 376 Pa. Nevertheless, this still allows the aerogel to regain its shape after removing the load. Based on Figure 6b, the calculated modulus of elasticity for the original sample is 44 kPa.

## 4. Conclusions

We developed a novel approach to the synthesis of aerogel-like materials via a one-pot process including surfactant-free synthesis of microcapsules from partially PEGylated hyperbranched polymethylethoxysiloxane and subsequent cross-linking of microcapsules with the residual hyperbranched polymer, yielding porous bulk material. Due to the highly diphilic nature of the PEGylated PMEOS, it readily concentrates at the oil/water interface in aqueous emulsions encapsulating oil droplets. During the subsequent condensation, the organosilicon precursor is consumed for oil encapsulation (yielding hollow microcapsules) followed by the formation of a continuous porous condensed phase. Concurrently, methyl groups ensure the hydrophobicity of the resulting gels, which eliminates the need of using additional reagents for their hydrophobization. Obtained aerogels have low density (down to 0.015 g/cm^3^), decent specific surface area (up to 337 m^2^/g), while mechanical properties may be deemed satisfactory as they allow maintaining the shape and undergo some elastic deformation up to 12%.

## Figures and Tables

**Table 1:** Loadings for the synthesis of PMEOS-PEG-5, 10 and 20.

Product	Ethoxy Group Substitution (mol%)	Mass of PMEOS (g)	Mass of PEG (g)	Yield (%)—(g)
PMEOS-PEG-5	5	40	4.5	(99%)—43.7
PMEOS-PEG-10	10	40	9	(99%)—47.5
PMEOS-PEG-20	20	40	18	(99%)—55.2

**Table 2:** IFT between toluene solution of PMEOS-PEG and water.

Sample	Concentration of PMEOS-PEG in Toluene (%)	IFT (mN/m)
PMEOS-PEG-5	0.1	20.5
PMEOS-PEG-5	1	12.2
PMEOS-PEG-5	5	11.2
PMEOS-PEG-5	10	10
PMEOS-PEG-10	0.1	16.2
PMEOS-PEG-10	1	9.7
PMEOS-PEG-10	5	9.2
PMEOS-PEG-10	10	8.5
PMEOS-PEG-20	0.1	14
PMEOS-PEG-20	1	7.1
PMEOS-PEG-20	5	5.7
PMEOS-PEG-20	10	5.2

**Table 3:** IFT between PMEOS-PEG dispersion in water and hexane.

Sample	Concentration of PMEOS-PEG in Water (%)	IFT (mN/m) ^1^
PMEOS-PEG-5	0.1	12.5
PMEOS-PEG-5	1	10
PMEOS-PEG-5	5	-
PMEOS-PEG-10	0.1	10.2
PMEOS-PEG-10	1	7
PMEOS-PEG-10	5	-
PMEOS-PEG-20	0.1	6.6
PMEOS-PEG-20	1	4.9
PMEOS-PEG-20	5	1.9

^1^ Due to the fact that the interfacial tension evaluation method is optical, investigating the surface activity of PMEOS-PEG-5 and PMEOS-PEG-10 at their 5% concentration in water was not feasible due to the excessive turbidity of these mixtures.

**Table 4:** Properties of obtained aerogels.

		Before Annealing		After Annealing ^1^			
Sample	PMEOS-PEG-20:Hexane	Density, g/cm^3^	Contact Angle, ^°^	Mass Loss, %	Density, g/cm^3^	Contact Angle, ^°^	Specific Surface Area (BET), m^2^/g
1	1:1	0.038	120 ± 7	59	0.015	140 ± 2	122 ± 7
2	2:1	0.040	120 ± 7	53	0.021	140 ± 2	337 ± 17
3	3:1	0.059	120 ± 7	55	0.026	140 ± 2	244 ± 12

^1^ Samples were annealed at 200 °C for 2 h.

## Data Availability

The original data reported in this study are available from the corresponding author on reasonable request.
